# Novel insights into diminished cardiac reserve in non-obstructive hypertrophic cardiomyopathy from four-dimensional flow cardiac magnetic resonance component analysis

**DOI:** 10.1093/ehjci/jead074

**Published:** 2023-04-28

**Authors:** Z Ashkir, S Johnson, A J Lewandowski, A Hess, E Wicks, M Mahmod, S Myerson, T Ebbers, H Watkins, S Neubauer, C J Carlhäll, B Raman

**Affiliations:** Oxford Centre for Clinical Magnetic Resonance Research (OCMR), Division of Cardiovascular Medicine, Radcliffe Department of Medicine, University of Oxford, John Radcliffe Hospital, Headington, Oxford OX3 9 DU, UK; Oxford Centre for Clinical Magnetic Resonance Research (OCMR), Division of Cardiovascular Medicine, Radcliffe Department of Medicine, University of Oxford, John Radcliffe Hospital, Headington, Oxford OX3 9 DU, UK; Oxford Cardiovascular Clinical Research Facility (CCRF), Division of Cardiovascular Medicine, Radcliffe Department of Medicine, University of Oxford, John Radcliffe Hospital, Headington, Oxford OX3 9 DU, UK; Wellcome Centre for Integrative Neuroimaging, Nuffield Department of Clinical Neurosciences (NDCN), University of Oxford, John Radcliffe Hospital, Headington, Oxford OX3 9 DU, UK; Oxford Centre for Clinical Magnetic Resonance Research (OCMR), Division of Cardiovascular Medicine, Radcliffe Department of Medicine, University of Oxford, John Radcliffe Hospital, Headington, Oxford OX3 9 DU, UK; Inherited Cardiovascular Conditions (ICC) Service, Oxford University Hospitals NHS Foundation Trust and the University of Oxford, Level 6, West Wing, John Radcliffe Hospital, Headington, Oxford OX3 9 DU, UK; Oxford Centre for Clinical Magnetic Resonance Research (OCMR), Division of Cardiovascular Medicine, Radcliffe Department of Medicine, University of Oxford, John Radcliffe Hospital, Headington, Oxford OX3 9 DU, UK; Oxford Centre for Clinical Magnetic Resonance Research (OCMR), Division of Cardiovascular Medicine, Radcliffe Department of Medicine, University of Oxford, John Radcliffe Hospital, Headington, Oxford OX3 9 DU, UK; Division of Diagnostics and Specialist Medicine, Department of Health, Medicine and Caring Sciences, Linköping University, SE-581 83 Linköping, Sweden; Center for Medical Image Science and Visualization (CMIV), Linköping University, SE-581 83 Linköping, Sweden; Division of Cardiovascular Medicine, Radcliffe Department of Medicine, University of Oxford, John Radcliffe Hospital, Headington, Oxford OX3 9 DU, UK; Oxford Centre for Clinical Magnetic Resonance Research (OCMR), Division of Cardiovascular Medicine, Radcliffe Department of Medicine, University of Oxford, John Radcliffe Hospital, Headington, Oxford OX3 9 DU, UK; Division of Diagnostics and Specialist Medicine, Department of Health, Medicine and Caring Sciences, Linköping University, SE-581 83 Linköping, Sweden; Center for Medical Image Science and Visualization (CMIV), Linköping University, SE-581 83 Linköping, Sweden; Department of Clinical Physiology in Linköping, Department of Health, Medicine and Caring Sciences, Linköping University, SE-581 83 Linköping, Sweden; Oxford Centre for Clinical Magnetic Resonance Research (OCMR), Division of Cardiovascular Medicine, Radcliffe Department of Medicine, University of Oxford, John Radcliffe Hospital, Headington, Oxford OX3 9 DU, UK

**Keywords:** hypertrophic cardiomyopathy, 4D-flow CMR, cardiac function, cardiac reserve

## Abstract

**Aims:**

Hypertrophic cardiomyopathy (HCM) is characterized by hypercontractility and diastolic dysfunction, which alter blood flow haemodynamics and are linked with increased risk of adverse clinical events. Four-dimensional flow cardiac magnetic resonance (4D-flow CMR) enables comprehensive characterization of ventricular blood flow patterns. We characterized flow component changes in non-obstructive HCM and assessed their relationship with phenotypic severity and sudden cardiac death (SCD) risk.

**Methods and results:**

Fifty-one participants (37 non-obstructive HCM and 14 matched controls) underwent 4D-flow CMR. Left-ventricular (LV) end-diastolic volume was separated into four components: direct flow (blood transiting the ventricle within one cycle), retained inflow (blood entering the ventricle and retained for one cycle), delayed ejection flow (retained ventricular blood ejected during systole), and residual volume (ventricular blood retained for >two cycles). Flow component distribution and component end-diastolic kinetic energy/mL were estimated. HCM patients demonstrated greater direct flow proportions compared with controls (47.9 ± 9% vs. 39.4 ± 6%, *P* = 0.002), with reduction in other components. Direct flow proportions correlated with LV mass index (*r* = 0.40, *P* = 0.004), end-diastolic volume index (*r* = −0.40, *P* = 0.017), and SCD risk (*r* = 0.34, *P* = 0.039). In contrast to controls, in HCM, stroke volume decreased with increasing direct flow proportions, indicating diminished volumetric reserve. There was no difference in component end-diastolic kinetic energy/mL.

**Conclusion:**

Non-obstructive HCM possesses a distinctive flow component distribution pattern characterised by greater direct flow proportions, and direct flow-stroke volume uncoupling indicative of diminished cardiac reserve. The correlation of direct flow proportion with phenotypic severity and SCD risk highlight its potential as a novel and sensitive haemodynamic measure of cardiovascular risk in HCM.

## Graphical Abstract

**Graphical Abstract:**
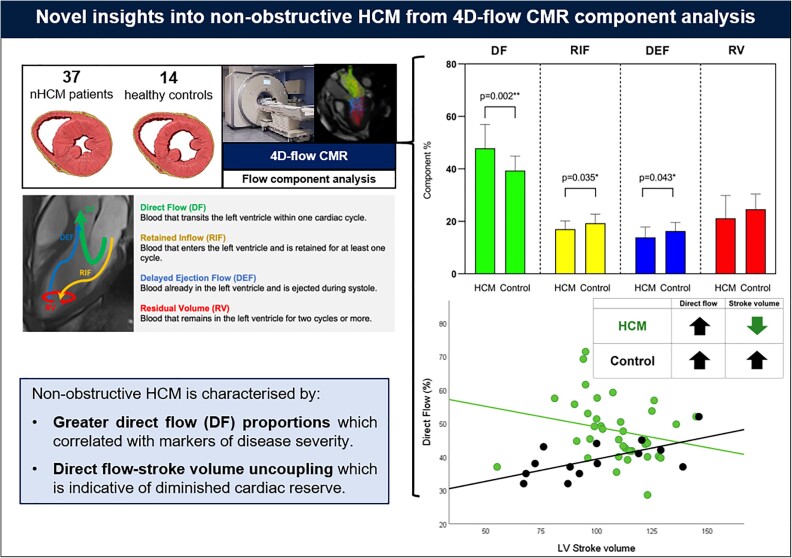

## Introduction

The paradoxical U-shaped relationship between left ventricular ejection fraction (LVEF) and mortality is increasingly recognized in population studies,^1–3^ but remains poorly understood. Hypertrophic cardiomyopathy (HCM) serves as an exemplar of this phenomenon, as LVEF is frequently supranormal in patients, yet mortality risk is elevated. Whilst accompanying haemodynamic disturbances such as left ventricular outflow (LVOT), obstruction and diastolic dysfunction^2^ generally mediate risks in HCM, such features may be absent in some individuals, such as in those with non-obstructive HCM.^3^ Conventional Doppler echocardiography is typically reserved for assessment of diastolic function in these patients, but has limited sensitivity for subtle changes in systolic blood flow. Consequently, there is an unmet need for more refined measures of ventricular haemodynamics in non-obstructive HCM, to enhance risk prediction and clarify the mechanisms underlying this U-shaped relationship between LVEF and outcomes.

Four-dimensional flow cardiac magnetic resonance (4D flow CMR) imaging is an emerging technology that overcomes many limitations of traditional imaging modalities. Through an assessment of velocity (and by extension flow) at any point in a three-dimensional (3D) data volume,^4,5^ 4D flow CMR allows for a comprehensive evaluation of left ventricular (LV) haemodynamics. There is a growing body of evidence in support of the role of 4D flow CMR in detecting subtle flow abnormalities in a variety of cardiovascular diseases.^6–10^

**Figure 1:**
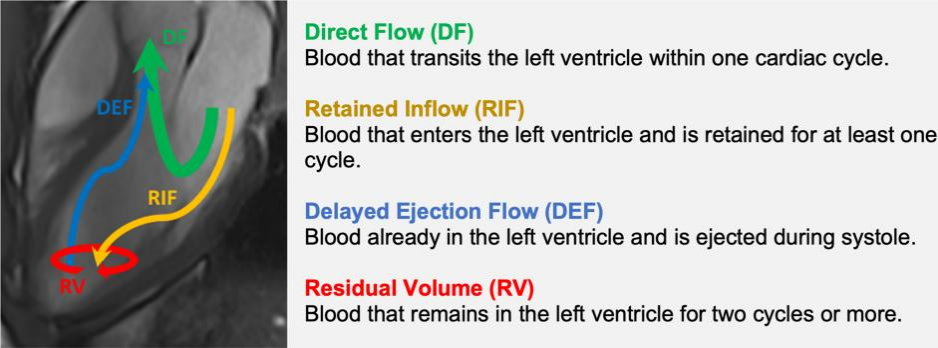
Constituent functional flow components of left ventricular blood volume.

Of the many analytical tools available, flow component analysis has gained significant traction for its ability to separate the left ventricular end-diastolic volume (LVEDV) into four distinct and functionally relevant components:^11–14^ direct flow (DF)—the most efficient component of ventricular blood, which transits the heart in one cardiac cycle, retained inflow (RIF)—blood that enters the LV during diastole but is retained for at least one cycle, delayed ejection flow (DEF)—blood already in the LV during diastole and which leaves during systole, and residual volume (RV)—blood that remains in the LV for at least two cycles (*Figure 1*).^15^

The relative distribution of these components and their kinetic energy provide a measure of diastolic–systolic coupling and flow efficiency.^10,11^ Previous studies have shown the importance of both ejected (DF and DEF) and non-ejected components (RV and RIF) in predicting exercise intolerance and thrombotic risks in cardiac diseases (e.g. in dilated and ischaemic cardiomyopathies^10,14–17^). Component end-diastolic kinetic energy (i.e. the kinetic energy of blood before systole or ‘presystolic momentum’) has also been identified as a novel marker of flow efficiency.^18^

To date, only a handful of studies have assessed haemodynamic changes in HCM using 4D flow CMR. Impaired vortex generation,^19^ higher ventricular flow velocities,^20^ increased outflow tract gradients, and energy losses^22^ have been noted in primarily obstructive HCM. None of these studies evaluated haemodynamic perturbations in non-obstructive HCM, and the risk associated with hypercontractility remains poorly explained.^1–3^

Here, we aimed to characterize the distribution of flow components on 4D flow CMR and end-diastolic kinetic energy (EF KE) in non-obstructive HCM and establish their relationship with phenotypic severity, sarcomeric mutation status, and estimated risk of sudden cardiac death (SCD) as per the European Society of Cardiology (ESC).^21^

## Methods

### Study population

Forty-five genotyped non-obstructive HCM patients were prospectively enrolled from the inherited cardiac conditions clinic at the John Radcliffe Hospital in Oxford, UK. Genetic screening using a 13-HCM gene panel testing was undertaken by the UKAS-accredited Oxford Medical Genetics Laboratory, and HCM was diagnosed based on LV wall thickness ≥15 mm (or ≥13 mm in genotype-positive patients) on CMR. Extensive quality control of 4D flow CMR datasets was undertaken, and 37 patient datasets were included in the final study analysis. We excluded HCM patients with left ventricular outflow tract (LVOT) obstruction (at rest or on provocation), previous myectomy, and those with MRI contraindications. Patients with significant cardiovascular co-morbidities such as severe hypertension, significant valvular heart disease or ischaemic heart disease were also excluded.

Fourteen healthy control subjects of similar age and gender, with no background of significant cardiac disease, a normal 12-lead electrocardiogram (ECG), and no family history of cardiomyopathy were enrolled for comparison.

The study was approved by the National Research Ethics Committee (REC ref 12/LO/1979). All participants provided informed written consent.

### Echocardiographic assessment

All participants underwent 2D transthoracic echocardiography using a Phillips EPIQ7 ultrasound system (Phillips, Netherlands) to assess diastolic function and LVOT gradients. Pulsed wave Doppler was used to measure trans-mitral early (*E*) and late (*A*) diastolic filling velocities, *E*/*A* ratio, and *E*-wave deceleration time. Pulsed tissue Doppler imaging was used to acquire mitral annular velocities at early diastolic filling (septal, lateral, and average *e′*). Left atrial (LA) size was also evaluated.

### CMR data acquisition

CMR scans were performed using a 3T Siemens Trio scanner (Siemens Healthcare, Erlangen, Germany) with a 32-channel cardiac surface coil. Morphological long axis and a stack of short axis images were acquired using a steady state free precession sequence with retrospective cardiac gating and during end-expiratory breath holds. Images were typically acquired using the following settings: echo time 1.12 ms, repetition time 35–40 ms, flip angle 50°, and slice thickness 8 mm. The field of view was adjusted for each subject to fully encompass the heart. Late gadolinium enhancement imaging was performed in all subjects according to standard clinical protocols.^23^

4D flow imaging was acquired using a free-breathing, retrospective ECG-triggered, and respiratory-gated sequence. Common acquisition parameters for 4D flow scanning were: velocity encoding 100–140 cm/s, repetition time 8 ms, echo time 2.5–3.0 ms, and flip angle 7°. The acquired spatial resolution was 3.0 × 3.0 × 3.0 mm^2^, and temporal resolution was 52 ms.

### Data analysis

#### CMR data analysis

LV volumetric, functional, and fibrosis analysis was performed using cvi42 (Circle Cardiovascular Imaging, Inc, Calgary, Canada) as previously described.^24^

The 4D flow data were analysed using a previously validated method by Eriksson *et al*.^11^ Briefly, two LV volume masks were created from endocardial segmentation of the morphological short axis stack at two time points between systole and diastole (at isovolumetric relaxation and contraction). The isovolumetric contraction LV volume was resampled to match the 4D CMR data resolution, and pathlines were emitted from the centre of each voxel and traced both forwards and backwards in time to cover systole and diastole. Pathlines were then automatically separated into DF, RIF, DEF, and RV. A previous study has established 4D flow component and KE analyses as highly repeatable and reproducible in healthy controls.^12^ In this study, we additionally evaluated inter-observer and intra-observer reliability among HCM patients (see Supplementary data online). Inter-observer correlation coefficient ranging from 0.86 to 0.96 across the different flow components and intra-observer correlations coefficients of 0.93–0.98 were noted across different flow components.

As part of quality control, datasets with ≥15% discrepancy between inflow and outflow volumes were excluded, as were those with non-physiological flow (e.g. pathlines that defied anatomical boundaries). As a result, eight HCM cases were excluded from analysis. For each component, KE/mL was calculated throughout the cardiac cycle using the equation KE (µJ/mL) = ½ × Mass × Velocity^2^ (where mass = mean density of blood (1060 kg/m^3^) × voxel volume).

### Statistical analysis

Statistical analyses were performed using SPSS Version 27.0 (IBM, Armonk, NY, USA). Normality was determined using the Kolmogorov–Smirnov test. Parametric continuous variables were presented using mean and SD, and non-parametric variables with median and interquartile range. Categorical data were described using frequency and percentages. Differences between cohorts were assessed using either the Kruskall–Wallis Test or Analysis of variance ANOVA (with *post hoc* Bonferroni correction) as appropriate. Associations between categorical variables were determined using the Chi-Square Test for independence or the Fischer’s Exact Test. Correlations between continuous variables were analysed using Pearson’s correlation coefficient for parametric data and Spearman’s correlation coefficient for non-parametric data. Statistical significance was set at *P* < 0.05.

## Results

### Participant characteristics

Demographic and clinical data are shown in *Table 1*. There were no significant differences in age and sex between HCM patients and controls. Twenty-five patients (68%) in the HCM group had a recognized pathogenic sarcomeric mutation.

**Table 1:** Baseline demographic and clinical parameters

	HCM (*n* = 37)	Control (*n* = 14)	*P* value
Age	50 ± 13	44 ± 20	0.248
Gender			
Male (%)	31 (84)	10 (71)	
Female (%)	6 (16)	4 (29)	0.321
BMI (kg/m^2^)	26 ± 3	23 ± 3	0.055
BSA (m^2^)	2 ± 0.2	1.9 ± 0.2	0.056
Sarcomeric mutation (%)	24 (65)	0	
Beta blocker/CCB use (%)	13 (35)	0	0.027
Hypertension (%)	2 (5)	1 (7)	0.814
HR (BPM)	64 ± 24	65 ± 11	0.836
E/A	1.2 ± 0.6	1.3 ± 0.4	0.709
Average E/e’	9.3 ± 5.3	7.3 ± 1.5	0.218
LA diameter (mm)	36 ± 6	30 ± 5	0.005**
LA EF (%)	56 (10)	57 (11)	0.177
LA SVI (mml/m^2^)	25 (11)	23 (10)	0.191
Maximum LV wall thickness (mm)	22 ± 5	11 ± 1	<0.001***
LVEDV (ml)	160 ± 28	160 ± 47	0.981
LVEDVI (mL/m^2^)	80 ± 13	86 ± 23	0.287
LVESV (ml)	51 ± 18	59 ± 25	0.172
LVESVI (mL/m^2^)	25 ± 8	32 ± 12	0.035*
LVSV (ml)	109 ± 17	100 ± 26	0.180
LVSVI (mL/m^2^)	55 ± 8	54 ± 13	0.790
LV EF (%)	69 ± 7	63 ± 7	0.016*
LV mass (g)	178 (58)	96 (39)	<0.001***
LV mass index (g/m^2^)	72 (23)	50 (13)	0.001**
LGE (g)	7 (10)	0	—

BMI, body mass index; BPM, beats per minute; CCB, calcium channel blocker; HR, heart rate; LA, left atrial; LV, left ventricle; LVED, left ventricular end-diastolic volume; LVEDVI, left ventricular end-diastolic volume index; LVESV, left ventricular systolic volume; LVEF, left ventricular ejection fraction; LGE, late gadolinium enhancement; LA SVI, LA stroke volume index.

Data presented as mean ± SD or median (interquartile range).

*P* < 0.05, **P < 0.01, ***P < 0.001.

As expected, HCM patients had significantly greater LV wall thickness (22 mm vs. 11 mm, *P* < 0.001), LV mass index (72 g/m^2^ vs. 50 g/m^2^, *P* = 0.001), and ejection fraction (EF) (69% vs. 63%, *P* = 0.016) compared with controls. LV end-diastolic volume index (LVEDVI) left ventricular end-systolic volume index (LVESVI) and stroke volume (LVSV) tended to be lower in the HCM group, however, this was not statistically significant in the case of LVEDVI and LVSV. As expected HCM patients had a greater LA diameter relative to controls (36 ± 6 mm vs. 30 ± 5 mm; *P* = 0.005), however, LA EF and LA stroke volume index were comparable with controls. There was no significant difference in conventional echocardiography based diastolic parameters between the groups. Within the HCM cohort, sarcomere mutation-positive (SARC+) and negative (SARC−) patients were comparable in baseline demographic and clinical characteristics apart from indexed LVEDVI, which was significantly smaller in the SARC- group (*Table 2*). The mean ESC SCD risk score in the HCM cohort was 2.4%.

**Table 2:** Baseline demographic and clinical parameters in sarcomere mutation-positive (SARC+) and negative (SARC−) patients

	SARC + (*n* = 25)	SARC- (*n* = 9)	*P* value
Age	50 ± 13	53 ± 15	0.565
Gender			
Male (%)	19 (76)	9 (100)	
Female (%)	6 (24)	0	0.105
BMI (kg/m^2^)	26 ± 4	28 ± 3	0.149
BSA (m^2^)	2 ± 0.2	2 ± 0.2	0.077
Βeta blocker/CCB use (%)	7 (28)	5 (55)	0.138
Hypertension (%)	1 (4)	1 (11)	0.437
HR (BPM)	58 ± 9	62 ± 14	0.334
*E*/*A*	1.3 ± 0.6	1.1 ± 0.3	0.594
Average *E*/*e′*	8.2 (3.5)	8.2 (3.9)	0.869
LA diameter (mm)	36 ± 7	35 ± 4	0.707
LA EF (%)	55 (11)	53 (15)	0.701
LA SVI (mml/m^2^)	25 (9)	25 (9)	0.939
Maximum LV wall thickness (mm)	19 (8)	19 (5)	0.878
LVEDV (ml)	163 ± 28	145 ± 28	0.113
LVEDVI (mL/m^2^)	83 ± 10	69 ± 10	0.001**
LVESV (ml)	53 ± 19	40 ± 14	0.064
LVESVI (mL/m^2^)	28 ± 8	20 ± 5	0.021*
LVSV (ml)	110 ± 15	103 ± 23	0.314
LVSVI (mL/m^2^)	56 (8)	53 (8)	0.079
LV EF (%)	68 ± 7	72 ± 7	0.168
LV Mass (g)	148 (60)	133 (64)	0.673
LV Mass index (g/m^3^)	69 (24)	72 (23)	0.908
LGE (g)	8 (11)	4 (5)	0.848

BMI, body mass index; BPM, beats per minute; CCB, calcium channel blocker; HR, heart rate; LA, left atrial; LV, left ventricle; LVED, left ventricular end-diastolic volume; LVEDVI, left ventricular end-diastolic volume index; LVESV, left ventricular end-systolic volume; LVEF, left ventricular ejection fraction; LGE, late gadolinium enhancement.

Data presented as mean ± SD or median (interquartile range).

*P* < 0.05, **P < 0.01, ***P < 0.001.

### Flow component proportions in HCM relative to controls

**Figure 2:**
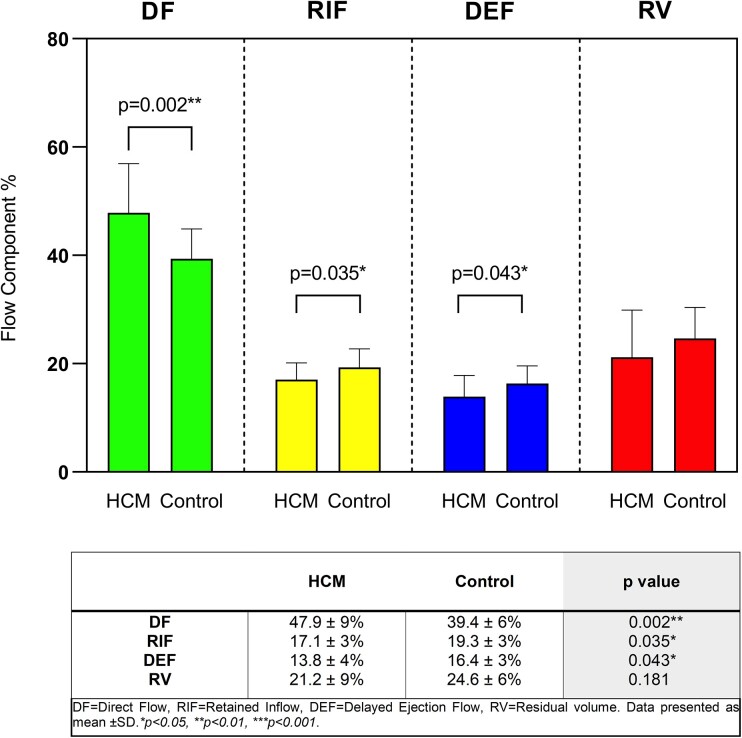
Distribution of flow components as percentage of left ventricular end diastolic volume (LVEDV) in HCM patients and controls, presented as mean ± sd. *P < 0.05, **P < 0.01.

There was a significant difference in flow component distribution between HCM patients and controls (*Figure 2*). HCM patients had significantly greater DF proportions compared with controls (47.9% vs. 39.4%, *P* = 0.002), lower RIF (17.1% vs. 19.3%, *P* = 0.035) and DEF (13.8% vs. 16.4%, *P* = 0.043), and comparable RV (21.2% vs. 24.6%, *P* = 0.181) proportions.

### Correlation between flow component proportions, cardiac phenotype and estimated risk of sudden cardiac death

**Figure 3:**
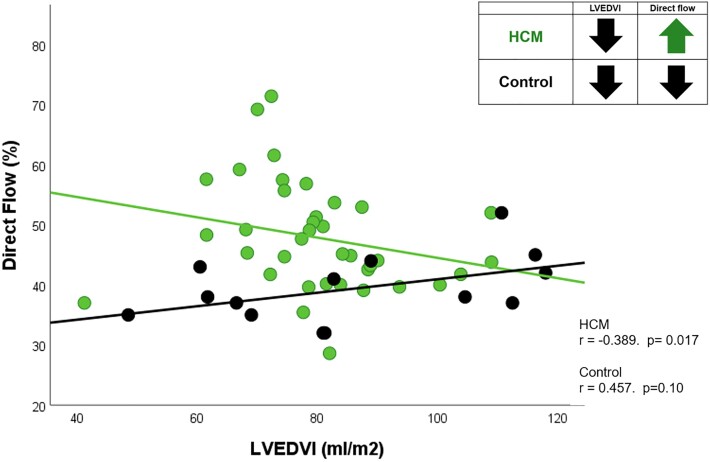
Scatter plot of direct flow (DF) proportion in HCM and controls against left ventricular end-diastolic volume index (LVEDVI).

In the entire study population (HCM and controls), DF proportions significantly increased with greater LV wall thickness (*r* = 0.370, *P* = 0.008) and LV mass index (*r* = 0.398 *P* = 0.004). In the HCM cohort alone, the positive correlation with LV mass index remained (*r* = 0.317, *P* = 0.056) and, additionally, HCM patients, unlike healthy controls, also demonstrated a significant negative correlation between DF proportion and LVEDVI (*r* = −0.389, *P* = 0.017) (i.e. the smaller the ventricular cavity, the higher the DF proportions) (*Figure 3*).

No relationship was seen between flow component proportions and extent of late gadolinium enhancement or diastolic function, but there was a significant positive association between DF proportions and the ESC risk score (*r* = 0.340, *P* = 0.039), which was not shared by LVEF (*r* = 0.232, *P* = 0.168), a more conventional haemodynamic CMR parameter. There was also a near significant difference in the DF% between patients with and without American Heart Association major risk factors^25^ for SCD (50% vs. 45%, *P* = 0.058).

### Contrasting relationship between stroke volume and flow component proportion in HCM and controls

**Figure 4:**
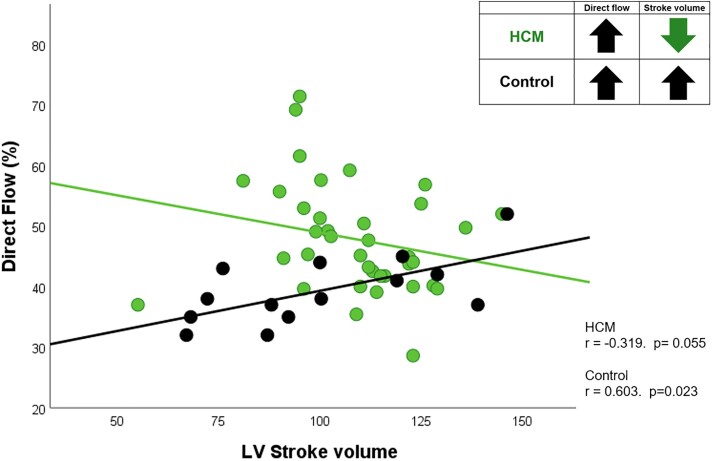
Scatter plot of direct flow (DF) proportion against stroke volume showing reduced stroke volume with increasing direct flow proportions in HCM patients (direct flow-stroke volume uncoupling) instead of the normal linear relationship seen in controls.

In controls, as the DF proportion increased, stroke volume was seen to sharply increase (*r* = 0.603, *P* = 0.023). By contrast, in HCM, as DF proportions increased, stroke volume tended to decrease among patients (*r* = −0.319. *P* = 0.055) (*Figure 4*). This inverse relationship was not present between LVEF and stroke volume in HCM patients.

### Differences in flow component proportions in HCM based on genotype

**Figure 5:**
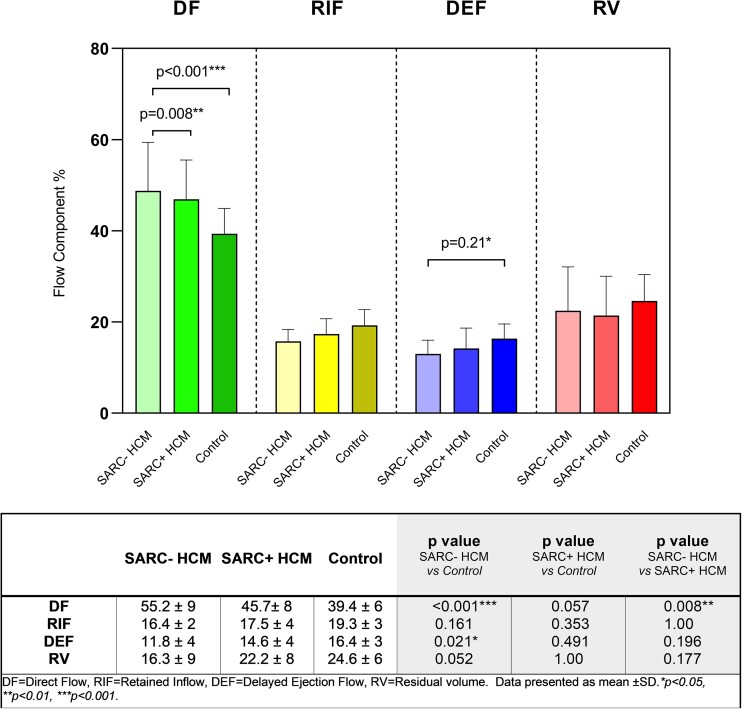
Distribution of flow components as percentage of left ventricular end diastolic volume (LVEDV) in HCM patients (categorized according to sarcomeric mutation status) and controls presented as mean ± sd. SARC+, sarcomeric mutation-positive; SARC−, sarcomeric mutation-negative. *P < 0.05, **P < 0.01, ***P < 0.001.

Categorization of HCM patients according to pathogenic sarcomeric mutation status yielded a difference in flow component distributions. In particular, DF proportions were significantly greater in sarcomeric mutation-negative HCM patients compared with sarcomere mutation-positive HCM patients (*P* = 0.008) (*Figure 5*), despite no significant difference in LV wall thickness (*P* = 0.878), LV mass index (*P* = 0.908), extent of late gadolinium enhancement (*P* = 0.848), or LA EF (*P* = 0.701). However, sarcomere mutation-negative HCM had significantly reduced LVEDVI when compared with mutation-positive HCM (*P* = 0.001)(*Table 2*).

### End diastolic kinetic energy in patients and controls

There was no observed difference in component ED KE/mL between HCM and controls, despite the increase in DF proportion (*Table 3*), nor when the HCM cohort was further divided according to sarcomeric mutation status or when mean diastolic KE/mL was measured. There was also no correlation between component ED KE/mL and maximum LV wall thickness, LV mass index, extent of fibrosis or LA size, LV volumes or function.

**Table 3:** Component end diastolic kinetic energy (ED KE)/mL

	HCM	Control	*P* value
DF ED KE (µJ/mL)	10 (5)	8 (7)	0.403
RIF ED KE (µJ/mL)	4 (2)	3 (3)	0.763
DEF ED KE (µJ/mL)	7 (6)	6 (6)	0.566
RV ED KE (µJ/mL)	2 (2)	2 (1)	0.384

DF, direct flow; RIF, retained inflow, DEF, delayed ejection flow; RV, residual volume.

Data presented as median (interquartile range).

## Discussion

This is the first study to comprehensively characterize ventricular blood flow component distribution and component KE using 4D-flow CMR in non-obstructive HCM patients, and to evaluate their relationship with cardiac phenotype and sarcomeric mutation status.

We found that, at rest, DF proportions are significantly higher in non-obstructive HCM compared with controls, in keeping with a hypercontractile left ventricle with a smaller cavity size. This increase in DF occurs at the expense of other components, with a significant reduction in RIF implying that a larger proportion of ventricular inflow is immediately converted into DF to maintain stroke volume. It is interesting to note that a similar pattern of flow component distribution has been previously described by Sundin *et al*.^26^ in healthy individuals following intravenous dobutamine administration. In their study, participants demonstrated a progressive increase in DF proportion and reciprocal reduction in other component proportions in response to inotropic stimulation. This increase in DF proportion was accompanied by a rise in stroke volume (SV) to maintain cardiac output in the setting of increased oxygen demand.

In our study, although non-obstructive HCM patients had a higher DF proportion than controls, there was an inverse relationship between DF proportion and stroke volume, sharply contrasting with the positive relationship between DF and stroke volumes in healthy subjects. Importantly, this relationship was not seen when assessing LVEF and stroke volume. We postulate that in non-obstructive HCM, ventricular hypertrophy and reduced cavity size favour greater DF proportions, and this means that less non-ejected blood can be accommodated. Since non-ejected components physiologically contribute to SV augmentation during conditions of increased workload (such as following dobutamine), we theorize that the inverse relationship between DF proportion and SV seen in HCM reflects diminished volumetric reserve in these patients. We speculate that this would also explain why HCM patients are at an increased risk of decompensation during sepsis,^27^ and the advantages of negative chronotropic agents (such as β-blockers), which prolong diastolic filling time. Additionally, this reduction in cardiac reserve could also explain the blunted stroke volume response sometimes observed in individuals with HCM during exercise.^28^ These findings are intriguing, and mirror haemodynamic changes seen in other models of hypertrophy and high ejection fraction. In a recent invasive haemodynamic study of HFpEF with LV hypertrophy, Rosch *et al*.^29^ demonstrated a reduction in stroke volume in response to handgrip exercise, particularly in those with high LVEF (>60%). The authors showed that both a reduction of preload reserve and increase in afterload were likely responsible for this observation. That flow component distributions can infer diminished volumetric reserve without the need for exercise or invasive measurements is noteworthy and warrants further investigation in other models of hypertrophy.

The correlation of DF with LV wall thickness and LV mass, and inverse relationship with LVEDVI in HCM suggests that DF proportions are closely linked to disease severity. This would also explain the association of flow components with estimated ESC SCD risk. Whether or not DF proportions at rest will offer another haemodynamic measure of risk like LVOT obstruction or blunted blood pressure response during exercise, remains to be investigated and would avoid the need for exercise provocation or invasive measurements to unmask reduced reserve.

In this study, sarcomere-negative HCM patients showed significantly greater DF proportions compared with sarcomere-positive HCM, suggesting a more marked derangement of normal function. While this could be a characteristic of sarcomere-negative HCM in general, it is possible that the sarcomere-negative HCM patients in our study had a more severe overall HCM phenotype than their sarcomere-positive counterparts. In keeping with this is the smaller indexed LV cavity size amongst the sarcomere-negative patients. Larger studies are needed to definitively assert a link between sarcomeric mutation status and distinct flow component profiles.

Component ED KE has generally been regarded as a measure of flow efficiency in health and disease. In patients with DCM^14,16,30^ and repaired tetralogy of Fallot,^31^ ED KE of the DF component was notably reduced and accompanied by a reduction in DF proportions. Similarly, in the study by Sundin *et al.*^26^ both DF ED KE and DF proportions were increased in healthy participants after administration of intravenous dobutamine. In the present study, DF ED KE was not different between HCM and controls, despite the increase DF proportions in HCM patients. We have considered two possible explanations to account for this. The first is that in the case of dobutamine, the physiological response not only involves an increase in contractility, but also a rise in heart rate and enhanced lusitropy, and these may contribute to greater KE preservation during diastole. The second is that the ventricle in HCM experiences greater energy losses in the form of viscous energy loss (due to greater wall friction), turbulent KE (due to rising intraventricular pressure), and/or disruption of the diastolic vortex (which can be compromised by a reduction in cavity size^32^). This may translate into lower pseudonormal end-diastolic KE despite a high DF proportion. Nonetheless, it is interesting to note that the flow component pattern in HCM is distinct to other conditions described in the literature and brings to light the complex pathophysiological processes at play in this disease.

### Study limitations

Although this is a small exploratory study and therefore underpowered for certain intergroup comparisons, we were able to probe the effects of hypertrophy on flow component analysis in non-obstructive HCM for the first time. Patients with LVOT obstruction were intentionally not included, and most of our non-obstructive HCM patients had no or only mild diastolic dysfunction indicating a mild phenotype. The generalizability of our findings to other HCM cohorts therefore may be limited. This could be viewed as a strength of this study, as it underscores the limited role of resting echocardiography in these patients and highlights the potential for 4D flow component analysis to further stratify non-obstructive HCM patients based on distinct haemodynamic characteristics.

### Clinical implications

Our work highlights the potential of 4D flow component analysis as an early imaging biomarker of risk in HCM, but further studies are needed to establish its prognostic value in the clinical setting. We provide mechanistic insights into the reduced volumetric reserve in HCM through a unique analysis of 4D flow CMR data. Multicentre studies involving a wide range of HCM patients (both in terms of genotype and phenotype) and validation against established invasive and non-invasive markers are still however needed to establish 4D flow component analysis as a clinically useful tool. Once this is achieved, its potential uses may be manifold, including differentiating physiological (athletic or exercise-induced) hypertrophy from pathological hypertrophy, and monitoring the effects of cardiac myosin inhibitors and the next generation of therapies in development (e.g. gene-editing therapies) in non-obstructive HCM where hypercontractility may not be sufficiently captured by assessment of LVEF alone.

## Conclusion

This study shows that patients with HCM possess a distinctive pattern of resting flow component distribution typified by greater direct flow proportions and direct flow-stroke volume uncoupling, in keeping with a diminished cardiac reserve. This appears to be associated with ventricular remodelling and provides additional insight into the mechanism of impaired stroke volume augmentation and U-shaped relationship between LV ejection fraction and mortality in patients. Comparison of HCM with and without sarcomeric mutations revealed less pronounced changes in direct flow proportions in sarcomere-positive patients, which may be driven by differences in severity of HCM phenotype. Collectively, our findings suggest that changes in the flow component distribution detect abnormal haemodynamic patterns missed by conventional echocardiography in non-obstructive HCM.

## Supplementary data

Supplementary data is available at *European Heart Journal - Cardiovascular Imaging* online.

## Supplementary Material

jead074_Supplementary_Data

## Data Availability

The data underlying this article will be shared on reasonable request to the corresponding author.

## References

[1] Wehner GJ , Jing L, Haggerty CM, Suever JD, Leader JB, Hartzel DN et al Routinely reported ejection fraction and mortality in clinical practice: where does the nadir of risk lie? Eur Heart J 2020;41:1249–57. 31386109 10.1093/eurheartj/ehz550 PMC8204658

[2] Curtis JP , Sokol SI, Wang Y, Rathore SS, Ko DT, Jadbabaie F et al The association of left ventricular ejection fraction, mortality, and cause of death in stable outpatients with heart failure. J Am Coll Cardiol 2003;42:736–42. 12932612 10.1016/s0735-1097(03)00789-7

[3] Toma M , Ezekowitz JA, Bakal JA, O’connor CM, Hernandez AF, Rizwan Sardar M et al The relationship between left ventricular ejection fraction and mortality in patients with acute heart failure: insights from the ASCEND-HF trial. Eur J Heart Fail 2014;16:334–41. 24464687 10.1002/ejhf.19

[4] Bogren HG , Mohiaddin RH, Yang GZ, Kilner PJ, Firmin DN. Magnetic resonance velocity vector mapping of blood flow in thoracic aortic aneurysms and grafts. J Thorac Cardiovasc Surg 1995;110:704–14. 7564437 10.1016/S0022-5223(95)70102-8

[5] Wigström L , Sjöqvist L, Wranne B. Temporally resolved 3D phase-contrast imaging. Magn Reson Med 1996;36:800–3. 8916033 10.1002/mrm.1910360521

[6] Schäfer M , Humphries S, Stenmark KR, Kheyfets VO, Buckner JK, Hunter KS et al 4D-flow Cardiac magnetic resonance-derived vorticity is sensitive marker of left ventricular diastolic dysfunction in patients with mild-to-moderate chronic obstructive pulmonary disease. Eur Heart J Cardiovasc Imaging 2018;19:415–24. 28460004 10.1093/ehjci/jex069 PMC6279084

[7] Crandon S , Westenberg JJM, Swoboda PP, Fent GJ, Foley JRJ, Chew PG et al Impact of age and diastolic function on novel, 4D flow CMR biomarkers of left ventricular blood flow kinetic energy. Sci Rep 2018;8:1–11. 30258186 10.1038/s41598-018-32707-5 PMC6158175

[8] Garg P , Crandon S, Swoboda PP, Fent GJ, Foley JRJ, Chew PG et al Left ventricular blood flow kinetic energy after myocardial infarction—insights from 4D flow cardiovascular magnetic resonance. J Cardiovasc Magn Reson 2018;20:1–15. 30165869 10.1186/s12968-018-0483-6 PMC6117925

[9] Fredriksson AG , Svalbring E, Eriksson J, Dyverfeldt P, Alehagen U, Engvall J et al 4D Flow MRI can detect subtle right ventricular dysfunction in primary left ventricular disease. J Magn Reson Imaging 2016;43:558–65. 26213253 10.1002/jmri.25015

[10] Svalbring E , Fredriksson A, Eriksson J, Dyverfeldt P, Ebbers T, Bolger AF et al Altered diastolic flow patterns and kinetic energy in subtle left ventricular remodeling and dysfunction detected by 4D flow MRI. PLoS One 2016;11:1–12. 10.1371/journal.pone.0161391 PMC4988651 27532640

[11] Eriksson J , Carlhäll C, Dyverfeldt P, Engvall J, Bolger A, Ebbers T. Semi-automatic quantification of 4D left ventricular blood flow. J Cardiovasc Magn Reson 2010;12:1–10. 20152026 10.1186/1532-429X-12-9 PMC2831022

[12] Stoll VM , Loudon M, Eriksson J, Bissell MM, Dyverfeldt P, Ebbers T et al Test-retest variability of left ventricular 4D flow cardiovascular magnetic resonance measurements in healthy subjects. J Cardiovasc Magn Reson 2018;20:1–10. 29499706 10.1186/s12968-018-0432-4 PMC5833126

[13] Bolger AF , Heiberg E, Karlsson M, Wigström L, Engvall J, Sigfridsson A et al Transit of blood flow through the human left ventricle mapped by cardiovascular magnetic resonance. J Cardiovasc Magn Reson 2007;9:741–7. 17891610 10.1080/10976640701544530

[14] Stoll VM, Hess AT, Rodgers CT, Bissell MM, Dyverfeldt P, Ebbers T *et al*. Left ventricular flow analysis: Novel imaging biomarkers and predictors of exercise capacity in heart failure. *Circ Cardiovasc Imaging*. 2019;**12**:e008130. 10.1161/CIRCIMAGING.118.008130 PMC6544522 31109184

[15] Ashkir Z, Myerson S, Neubauer S, Carlhäll C-J, Ebbers T, Raman B. Four-dimensional flow cardiac magnetic resonance assessment of left ventricular diastolic function. *Front Cardiovasc Med* 2022;**9**:194. 10.3389/fcvm.2022.866131 PMC9355735 35935619

[16] Eriksson J , Bolger AF, Ebbers T, Carlhäll CJ. Four-dimensional blood flow-specific markers of LV dysfunction in dilated cardiomyopathy. Eur Heart J Cardiovasc Imaging 2013;14:417–24. 22879457 10.1093/ehjci/jes159 PMC3626338

[17] Corrado PA , Macdonald JA, François CJ, Aggarwal NR, Weinsaft JW, Wieben O. Reduced regional flow in the left ventricle after anterior acute myocardial infarction: a case control study using 4D flow MRI. BMC Med Imaging 2019;19:1–10. 31888531 10.1186/s12880-019-0404-7 PMC6937788

[18] Eriksson J , Dyverfeldt P, Engvall J, Bolger AF, Ebbers T, Carlhäll CJ. Quantification of presystolic blood flow organization and energetics in the human left ventricle. Am J Physiol Heart Circ Physiol 2011;300:2135–41. 10.1152/ajpheart.00993.2010 21421820

[19] Martínez-Legazpi P , Bermejo J, Benito Y, Yotti R, Pérez Del Villar C, González-Mansilla A et al Contribution of the diastolic vortex ring to left ventricular filling. J Am Coll Cardiol. 2014;64:1711–21. 25323260 10.1016/j.jacc.2014.06.1205

[20] Pruijssen JT, Allen BD, Barker AJ, Bonow RO, Choudhury L, Carr JC et al. Hypertrophic cardiomyopathy is associated with altered left ventricular 3D blood flow dynamics. *Radiol Cardiothorac Imaging* 2020;**2**:e190038. 10.1148/ryct.2020190038 PMC7977986 33778534

[21] O'Mahony C , Jichi F., Pavlou M, Monserrat L., Anastasakis A, Rapezzi C et al A novel clinical risk prediction model for sudden cardiac death in hypertrophic cardiomyopathy (HCM Risk-SCD). Eur Heart J 2014;35:2010–2020. 10.1093/eurheartj/eht439 24126876

[22] van Ooij P , Allen BD, Contaldi C, Garcia J, Collins J, Carr J et al 4D Flow MRI and T1-mapping: assessment of altered cardiac hemodynamics and extracellular volume fraction in hypertrophic cardiomyopathy. J Magn Reson Imaging 2016;43:107–14. 26227419 10.1002/jmri.24962 PMC4850842

[23] Kellman P , Arai AE, McVeigh ER, Aletras AH. Phase-sensitive inversion recovery for detecting myocardial infarction using gadolinium-delayed hyperenhancement. Magn Reson Med 2002;47:372–83. 11810682 10.1002/mrm.10051 PMC2041905

[24] Raman B , Ariga R, Spartera M, Sivalokanathan S, Chan K, Dass S et al Progression of myocardial fibrosis in hypertrophic cardiomyopathy: mechanisms and clinical implications. Eur Heart J Cardiovasc Imaging 2019;20:157–67. 30358845 10.1093/ehjci/jey135 PMC6343081

[25] Members WC , Ommen SR, Mital S, Burke MA, Day SM, Deswal A et al 2020 AHA/ACC guideline for the diagnosis and treatment of patients with hypertrophic cardiomyopathy. Circulation 2020;105:207–9.

[26] Sundin J , Engvall J, Nylander E, Ebbers T, Bolger AF, Carlhäll CJ. Improved efficiency of intraventricular blood flow transit under cardiac stress: A 4D flow dobutamine CMR study. Front Cardiovasc Med 2020;7(November):1–10. 33324686 10.3389/fcvm.2020.581495 PMC7724031

[27] Poliac LC , Barron ME, Maron BJ. Hypertrophic cardiomyopathy. Anesthesiology 2006;104:183–92. 16394705 10.1097/00000542-200601000-00025

[28] Sadoul N , Prasad K, Elliott PM, Bannerjee S, Frenneaux MP, McKenna WJ. Prospective prognostic assessment of blood pressure response during exercise in patients with hypertrophic cardiomyopathy. Circulation 1997;96:2987–91. 9386166 10.1161/01.cir.96.9.2987

[29] Rosch S , Kresoja KP, Besler C, Fengler K, Schöber AR, von Roeder M et al Characteristics of heart failure with preserved ejection fraction across the range of left ventricular ejection fraction. Circulation 2022;146:506–18. 35862208 10.1161/CIRCULATIONAHA.122.059280

[30] Eriksson J , Bolger AF, Ebbers T, Carlhäll CJ. Assessment of left ventricular hemodynamic forces in healthy subjects and patients with dilated cardiomyopathy using 4D flow MRI. Physiol Rep 2016;4:1–12. 10.14814/phy2.12685 PMC4758930 26841965

[31] Zhao X , Hu L, Leng S, Tan RS, Chai P, Bryant JA et al Ventricular flow analysis and its association with exertional capacity in repaired tetralogy of Fallot: 4D flow cardiovascular magnetic resonance study. J Cardiovasc Magn Reson 2022;24:1–17. 34980199 10.1186/s12968-021-00832-2 PMC8722058

[32] Arvidsson PM , Kovács SJ, Töger J, Borgquist R, Heiberg E, Carlsson M et al Vortex ring behavior provides the epigenetic blueprint for the human heart. Sci Rep 2016;6:1–9. 26915473 10.1038/srep22021 PMC4768103

