# Supplementary material for: Novel insights into diminished cardiac reserve in non-obstructive hypertrophic cardiomyopathy from four-dimensional flow cardiac magnetic resonance component analysis
Source: Eur Heart J Cardiovasc Imaging. 2023 Apr 28;24(9):1192–200. doi: 10.1093/ehjci/jead074 (PMC10445247; doi:10.1093/ehjci/jead074)
Supplement: jead074_Supplementary_Data [file jead074_supplementary_data.docx]

**SUPPLEMENT**

**Interobserver variability for flow component distributions.**

| Flow component distribution results observer 1 (SJ) vs observer 2 (ZA) | | | | | | | | |
| --- | --- | --- | --- | --- | --- | --- | --- | --- |
| **CASE** | **DF%**  **(SJ)** | **RIF%**  **(SJ)** | **DEF%**  **(SJ)** | **RV%**  **(SJ)** | **DF%**  **(ZA)** | **RIF%**  **(ZA)** | **DEF%**  **(ZA)** | **RV%**  **(ZA)** |
| 1 | 36.64 | 25.33 | 12.55 | 25.48 | 41.28 | 24.41 | 12.90 | 21.41 |
| 2 | 57.59 | 17.84 | 13.42 | 11.15 | 60.21 | 18.15 | 12.14 | 9.49 |
| 3 | 34.53 | 21.81 | 17.67 | 25.98 | 36.01 | 21.48 | 18.03 | 24.47 |
| 4 | 40.01 | 20.12 | 19.71 | 20.16 | 38.56 | 19.69 | 20.47 | 21.29 |
| 5 | 48.54 | 18.29 | 13.16 | 20.01 | 47.44 | 18.48 | 14.61 | 19.48 |
| 6 | 61.57 | 18.22 | 11.67 | 8.54 | 60.92 | 19.83 | 11.08 | 8.17 |
| 7 | 39.67 | 13.74 | 16.64 | 29.94 | 46.64 | 12.72 | 15.72 | 24.92 |
| 8 | 49.74 | 14.17 | 13.46 | 22.64 | 48.56 | 15.19 | 13.31 | 22.95 |
| 9 | 31.52 | 31.92 | 12.56 | 24.00 | 40.75 | 25.11 | 13.88 | 20.26 |
| 10 | 59.21 | 20.31 | 10.13 | 10.35 | 59.14 | 22.09 | 8.96 | 9.81 |
| DF= Direct flow, RIF=Retained inflow, DEF=Delayed ejection flow, RV=Residual volume. | | | | | | | | |

| Inter-observer Correlation Results | | | | | | | |
| --- | --- | --- | --- | --- | --- | --- | --- |
| **DF%** | **p** | **RIF%** | **p** | **DEF%** | **p** | **RV%** | **p** |
| 0.93 | <0.001 | 0.86 | <0.001 | 0.95 | <0.001 | 0.96 | <0.001 |


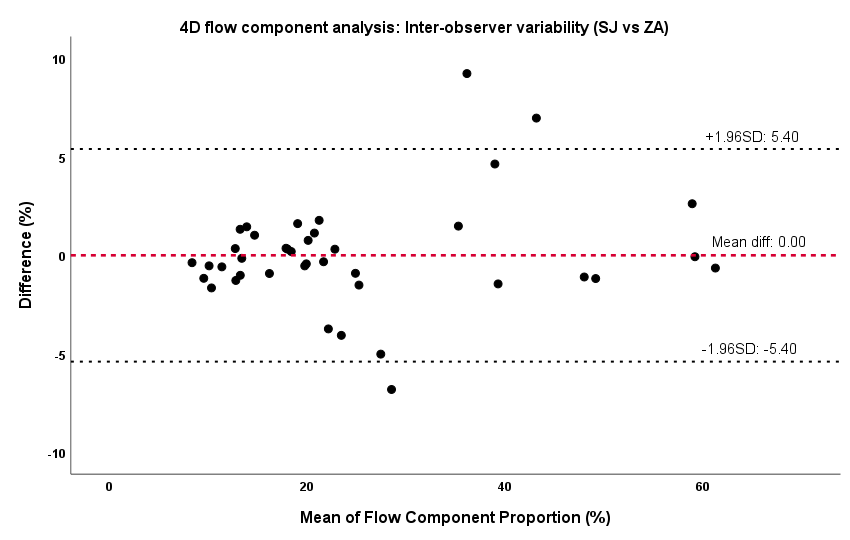


**Figure 1**. Bland-Altman plot demonstrating close inter-observer (SJ & ZA) agreement in flow component % measurements.

**Intra-observer variability**

| Flow component distribution results observer 2 x 2 | | | | | | | | |
| --- | --- | --- | --- | --- | --- | --- | --- | --- |
| **CASE** | **DF%**  **(ZA1)** | **RIF%**  **(ZA1)** | **DEF%**  **(ZA1)** | **RV%**  **(ZA1)** | **DF%**  **(ZA2)** | **RIF%**  **(ZA2)** | **DEF%**  **(ZA2)** | **RV%**  **(ZA2)** |
| 1 | 41.28 | 24.41 | 12.90 | 21.41 | 37.89 | 24.53 | 13.06 | 24.53 |
| 2 | 60.21 | 18.15 | 12.14 | 9.49 | 57.51 | 18.28 | 13.31 | 10.90 |
| 3 | 36.01 | 21.48 | 18.03 | 24.47 | 36.73 | 21.37 | 18.05 | 23.85 |
| 4 | 38.56 | 19.69 | 20.47 | 21.29 | 36.51 | 19.40 | 20.26 | 23.84 |
| 5 | 47.44 | 18.48 | 14.61 | 19.48 | 53.85 | 16.11 | 11.79 | 18.26 |
| 6 | 60.92 | 19.83 | 11.08 | 8.17 | 57.87 | 20.06 | 11.59 | 10.48 |
| 7 | 46.64 | 12.72 | 15.72 | 24.92 | 45.33 | 12.64 | 15.70 | 26.32 |
| 8 | 48.56 | 15.19 | 13.31 | 22.95 | 46.61 | 14.19 | 15.32 | 23.87 |
| 9 | 40.75 | 25.11 | 13.88 | 20.26 | 40.65 | 24.95 | 14.17 | 20.23 |
| 10 | 59.14 | 22.09 | 8.96 | 9.81 | 56.76 | 22.34 | 9.62 | 11.29 |
| DF= Direct flow, RIF=Retained inflow, DEF=Delayed ejection flow, RV=Residual volume. | | | | | | | | |

| Intra-observer Correlation Results | | | | | | | |
| --- | --- | --- | --- | --- | --- | --- | --- |
| **DF%** | **p** | **RIF%** | **p** | **DEF%** | **p** | **RV%** | **p** |
| 0.95 | <0.001 | 0.98 | <0.001 | 0.93 | <0.001 | 0.98 | <0.001 |


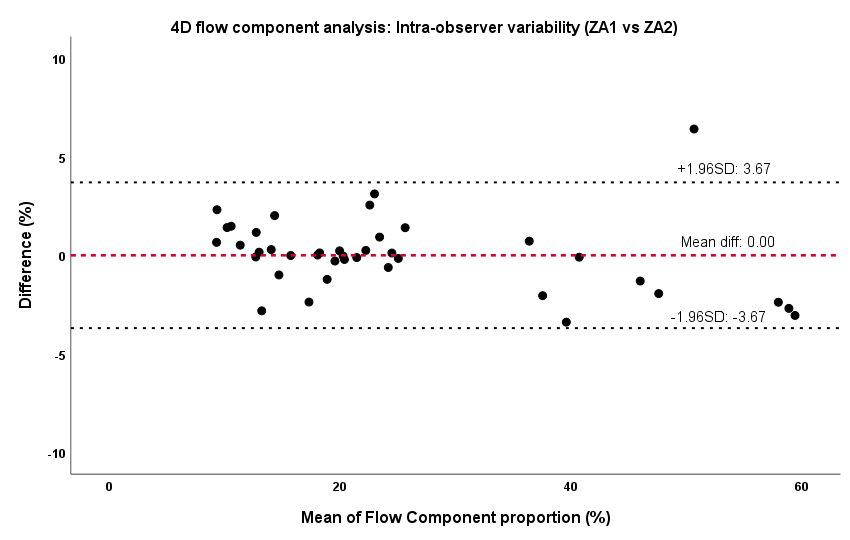


**Figure 2**. Bland-Altman plot demonstrating close intra-observer (ZA1 & ZA2) agreement in flow component % measurements.
